# Effects of *Thymus vulgaris* L., *Cinnamomum verum* J.Presl and *Cymbopogon nardus* (L.) Rendle Essential Oils in the Endotoxin-induced Acute Airway Inflammation Mouse Model

**DOI:** 10.3390/molecules25153553

**Published:** 2020-08-04

**Authors:** Eszter Csikós, Kata Csekő, Amir Reza Ashraf, Ágnes Kemény, László Kereskai, Béla Kocsis, Andrea Böszörményi, Zsuzsanna Helyes, Györgyi Horváth

**Affiliations:** 1 Department of Pharmacognosy, Faculty of Pharmacy, University of Pécs, H-7624 Pécs, Hungary; csikos.eszter@gytk.pte.hu (E.C.); amirreza@gmail.com (A.R.A.); 2 Department of Pharmacology and Pharmacotherapy, Medical School, University of Pécs, H-7624 Pécs, Hungary; csekoe.kata@gmail.com (K.C.); agnes.kemeny@aok.pte.hu (Á.K.); zsuzsanna.helyes@aok.pte.hu (Z.H.); 3 Szentágothai Research Centre, University of Pécs, H-7624 Pécs, Hungary; 4 Department of Medical Biology and Central Electron Microscope Laboratory, Medical School, University of Pécs, H-7624 Pécs, Hungary; 5 Department of Pathology, Medical School, University of Pécs, H-7624 Pécs, Hungary; kereskai.laszlo@pte.hu; 6 Department of Medical Microbiology and Immunology, Medical School, University of Pécs, H-7624 Pécs, Hungary; kocsis.bela@pte.hu; 7 Department of Pharmacognosy, Faculty of Pharmacy, Semmelweis University, H-1085 Budapest, Hungary; boszormenyi.andrea@pharma.semmelweis-univ.hu; 8 PharmInVivo Ltd., H-7629 Pécs, Hungary

**Keywords:** thyme, lemongrass, cinnamon, essential oil, endotoxin, airway inflammation, mouse model, airway hyperresponsiveness, myeloperoxidase activity, perivascular edema

## Abstract

Thyme (TO), cinnamon (CO), and Ceylon type lemongrass (LO) essential oils (EOs) are commonly used for inhalation. However, their effects and mechanisms on inflammatory processes are not well-documented, and the number of in vivo data that would be important to determine their potential benefits or risks is low. Therefore, we analyzed the chemical composition and investigated the activity of TO, CO, and LO on airway functions and inflammatory parameters in an acute pneumonitis mouse model. The components of commercially available EOs were measured by gas chromatography–mass spectrometry. Airway inflammation was induced by intratracheal endotoxin administration in mice. EOs were inhaled during the experiments. Airway function and hyperresponsiveness were determined by unrestrained whole-body plethysmography on conscious animals. Myeloperoxidase (MPO) activity was measured by spectrophotometry from lung tissue homogenates, from which semiquantitative histopathological scores were assessed. The main components of TO, CO, and LO were thymol, cinnamaldehyde, and citronellal, respectively. We provide here the first evidence that TO and CO reduce inflammatory airway hyperresponsiveness and certain cellular inflammatory parameters, so they can potentially be considered as adjuvant treatments in respiratory inflammatory conditions. In contrast, Ceylon type LO inhalation might have an irritant effect (e.g., increased airway hyperresponsiveness and MPO activity) on the inflamed airways, and therefore should be avoided.

## 1. Introduction

Essential oils (EOs) are made up of secondary metabolites by plants. They are liquid mixtures of various compounds (such as terpenoids and phenylpropane derivates) that evaporate at room temperature. Their most common extraction method is distillation. Due to their characteristic aroma and biological effects, EOs are commonly used for different purposes in the fields of aromatherapy, food preservation, plant pathology and medical microbiology. The application of EOs is increasing among patients as a single or adjuvant treatment for respiratory diseases and inflammations based on traditional use and empirical evidence.

Inflammatory lung diseases affect a large population at every age worldwide, and they also appear on the list of the top 10 causes of death [1]. These diseases are associated with acute or chronic inflammation and deteriorate the patients′ quality of life.

The advantages of EOs are that they can easily reach the respiratory tract via inhalation due to their volatility, and because of their complex composition they can exert multiple actions through the modification of lipoxygenase, cyclooxygenase and inducible nitric oxide synthase function as well as the inhibition of the inflammatory mediator production [2,3]. Recent studies have proved the anti-inflammatory effects of eucalyptus and lavender EOs, 1,8-cineole (eucalyptol), and menthol [2,4,5,6].

Thyme (TO), cinnamon (CO) and lemongrass (LO) EOs are commonly used via inhalation. Their components have been shown to reduce inflammatory cytokine production and some inflammatory parameters [7,8,9,10,11,12,13,14,15]. However, the results of previous studies are poorly comparable and not conclusive, because the studies used EOs of different sources, purity and compositions, mainly certain isolated components are studied, moreover, the experimental paradigms, investigated parameters and the outcomes greatly differ from each other. Furthermore, there are only few in vivo data and airway function results are even more scarcely available, which would be important to determine their potential clinical benefits and risks, particularly under inflammatory conditions.

TO is extracted from the fresh flowering aerial parts of *Thymus vulgaris* L. (Lamiaceae) and other *Thymus* species via steam distillation. *Thymus vulgaris* has several chemotypes containing different main components such as thymol, carvacrol, linalool, and geraniol. The most prevalent use of TO is by inhalation as an expectorant in bronchitis and common cold [16,17].

We used the EO of *Cinnamomum verum* J.S. Presl. (Lauraceae) as CO, isolated by steam distillation from the bark of the shoots with *trans*-cinnamaldehyde, as the main component. In medieval times, CO was used against frontal sinusitis and common cold, but nowadays, it is more commonly used for the treatment of gastrointestinal problems [18].

LO is obtained by steam distillation from the fresh or partially dried aerial parts of *Cymbopogon* species. We used the EO of *Cymbopogon nardus* (L.) Rendle (Poaceae, Ceylon type). Characteristic components are citronellal, geraniol, citronellol, limonene, citronellyl acetate, geranyl acetate and neral. The treatment of common cold is among the traditional uses of LO [19,20].

The endotoxin (lipopolysaccharide: LPS)-induced acute airway inflammation mouse model is the most commonly used, well-reproducible mechanism model for the study of EOs in acute respiratory inflammation [10,13,15,21]. LPS is a compound of the Gram-negative bacterial cell wall that causes interstitial pneumonitis and acute respiratory obstruction with a well-defined mechanism by activating Toll-like receptor-4 on macrophages, resulting in the release of several inflammatory mediators and consequent neutrophil activation after intranasal or intratracheal administration [22].

Here, we analyzed the chemical composition of TO, CO and LO, and investigated their effects on airway functions and inflammatory parameters in the endotoxin-induced acute pneumonitis mouse model, which is a characterized pathophysiological mechanism model relevant to several conditions of human lung inflammation.

## 2. Results

### 2.1. Chemical Analysis of EOs

Chemical analysis of EOs was conducted using gas chromatography with a flame-ionization detector (GC-FID) and GC-MS. The main components of TO were thymol (46.3%) and *p*-cymene (22.1%); cinnamaldehyde was the main component (74.0%) in CO; whereas citronellal (36.2%) and geraniol (25.3%) were present in the highest concentrations in LO (Table 1). Other significant identified compounds included 1,8-cineole (9.8%), linalool (5.1%), and carvacrol (3.2%) in TO; cinnamyl acetate (5.3%) and linalool (3.9%) in CO; and citronellol (13.6%), limonene (3.6%) in LO.

### 2.2. Respiratory Functions

**Figure 1:**
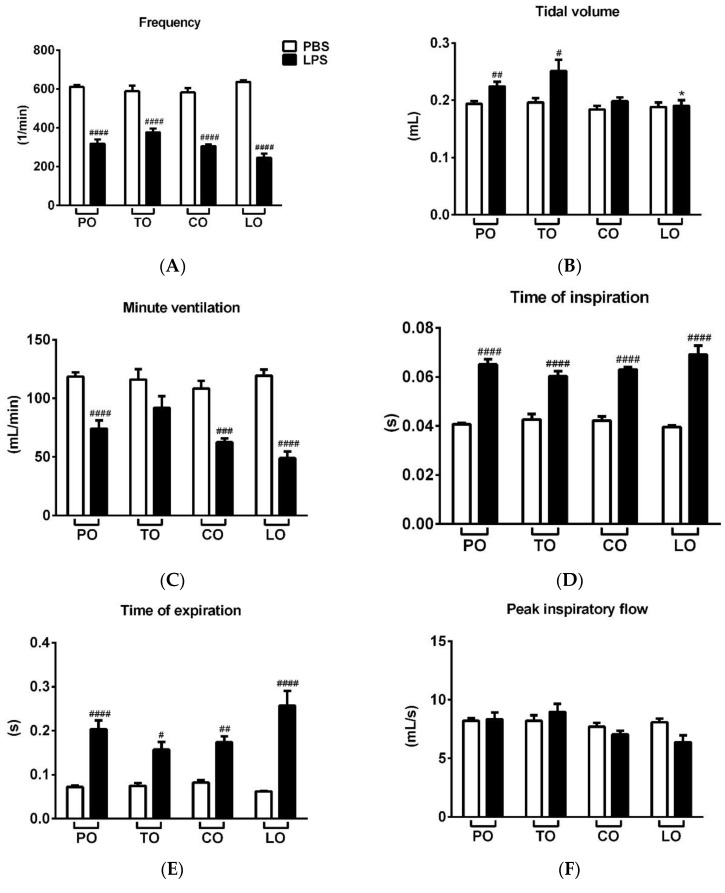

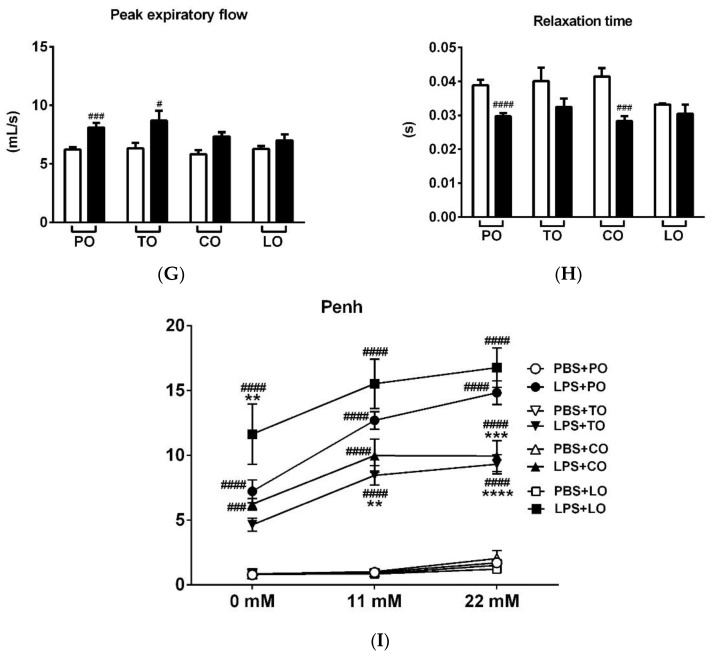
Effects of thyme (TO), cinnamon (CO) and lemongrass (LO) essential oils on (**A**) breathing frequency, (**B**) tidal volume, (**C**) minute ventilation, (**D**) time of inspiration, (**E**) time of expiration, (**F**) peak inspiratory flow, (**G**) peak expiratory flow, (**H**) relaxation time and (**I**) Penh compared to the negative control paraffin oil (PO), after lipopolysaccharide (LPS - black columns)/phosphate-buffered saline (PBS - white columns) treatment. (*n* = 8–10/group, # *p* < 0.05, ## *p* < 0.005, ### *p* < 0.0005, #### *p* < 0.0001 vs. respective PBS-treated group, *p < 0.05, ** *p* < 0.005, *** *p* < 0.0005, **** *p* < 0.0001 vs. LPS-PO-treated mice).

LPS treatment significantly reduced the frequency, minute ventilation and relaxation time and increased tidal volume, time of inspiration and expiration, as well as peak expiratory flow 24 h after administration, whereas it did not alter peak inspiratory flow (Figure 1A–H, Figures S1 and S2). Penh correlating with airway hyperresponsiveness was remarkably increased already at baseline measurement and was further aggravated upon carbachol inhalation-induced bronchoconstriction in LPS-treated mice. CO and TO inhalation significantly reduced the carbachol-induced hyperresponsiveness in LPS-treated mice compared to paraffin oil (PO) inhalation used as a negative control, whereas LO significantly deteriorated baseline Penh (Figure 1I and Figure S1). Regarding the other basal respiratory parameters, tidal volume was significantly reduced by LO compared to the PO-treated LPS-administered group, but this seemingly ameliorating effect could be the result of the lowest frequency and minute ventilation measured in all groups.

### 2.3. Lung Histopathological Evaluation

**Figure 2:**
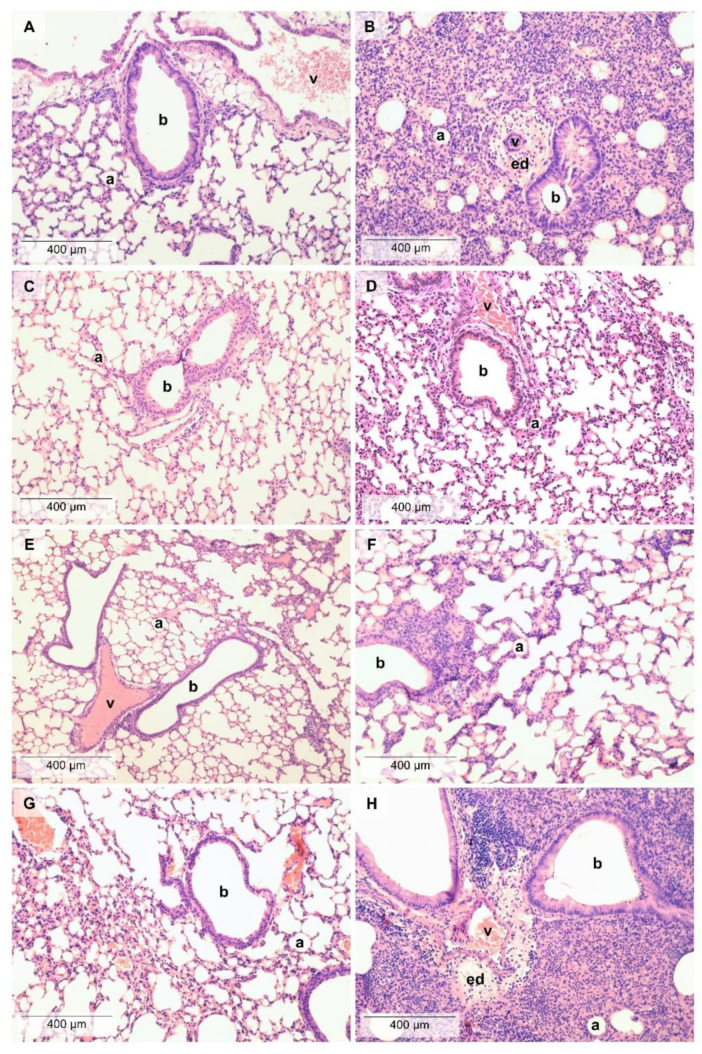
Histopathological alterations in the lung. Representative pictures of lung parenchyma after (**A**) PBS and PO treatment, (**B**) LPS and PO treatment, (**C**) PBS and TO treatment, (**D**) LPS and TO treatment, (**E**) PBS and CO treatment, (**F**) LPS and CO treatment, (**G**) PBS and LO treatment, and (**H**) LPS and LO treatment. (Hematoxylin–eosin staining, 200 × magnification; a: alveolus, b: bronchiole, v: vessel, ed: edema).

**Figure 3:**
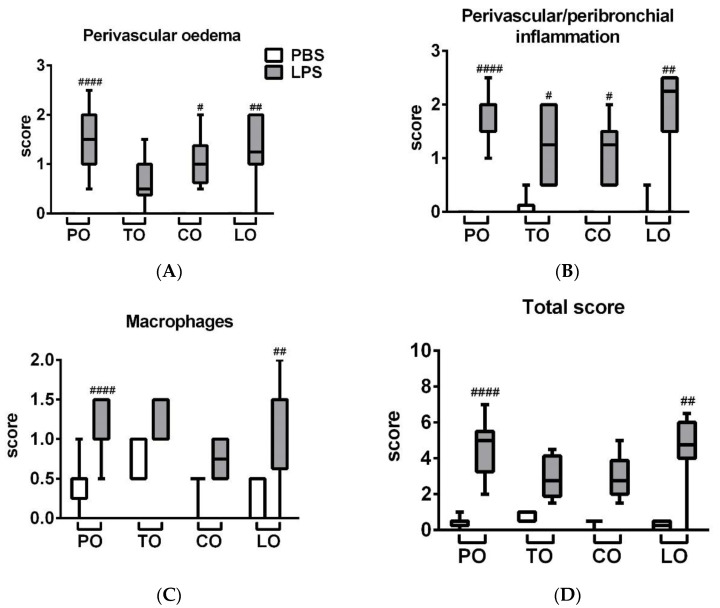
Semiquantitative evaluation of (**A**) perivascular and peribronchial edema, (**B**) accumulation of neutrophil granulocytes, (**C**) macrophages and (**D**) total score of lung histopathological alterations (*n* = 8–10/group, # *p* < 0.05, ## *p* < 0.005, #### *p* < 0.000 vs. respective PBS-treated group) are demonstrated in box plots showing the median, upper/lower quartile, and maximum/minimum values.

LPS administration induced a massive inflammatory infiltration in the lung composed of neutrophil granulocytes and macrophages associated with a remarkable perivascular and peribronchial edema formation (Figure 2). LPS-induced edema formation was decreased by TO, which was not significantly increased compared to its respective phosphate-buffered saline (PBS)-treated control. Regarding macrophage infiltration and total score, neither TO nor CO were significantly increased compared to their PBS-treated controls. LO did not influence the extent of histopathological alterations (Figure 3), although we found mild goblet cell hyperplasia, similarly to the LPS-treated group, and focal lymphocyte follicles after LO inhalation (data not shown).

### 2.4. Myeloperoxidase (MPO) Activity

**Figure 4:**
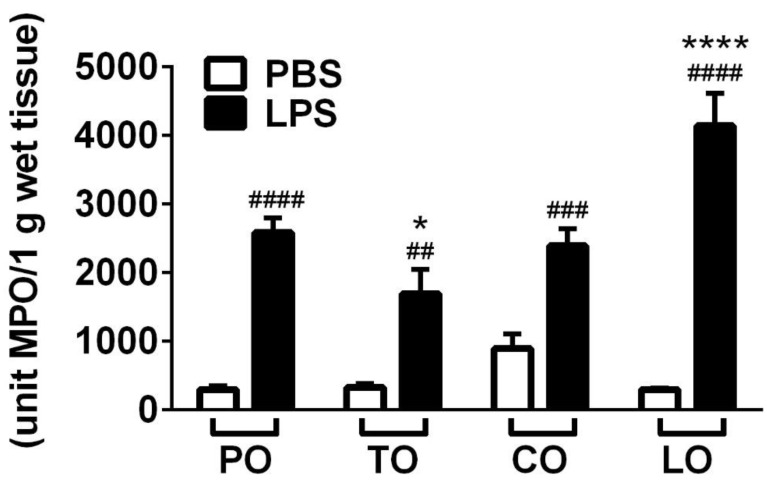
Results of myeloperoxidase (MPO) activity measurements. (*n* = 8–10/group, ## *p* < 0.005, ### *p* < 0.0005, #### *p* < 0.0001 vs. respective PBS-treated group, * *p* < 0.05, **** *p* < 0.0001 vs. LPS-PO-treated).

MPO activity—correlating with the number of activated granulocytes and macrophages—significantly increased after LPS treatment in the lung homogenates in accordance with the histopathological findings. TO decreased but, surprisingly, LO inhalation further increased enzyme activity in the LPS-treated animals, while CO had no significant effect on this parameter (Figure 4).

## 3. Discussion

We provide here the first evidence that TO (with thymol and *p*-cymene as main compounds) and CO (with cinnamaldehyde as main component) inhalation decrease inflammatory airway hyperresponsiveness and histopathological alterations in the endotoxin-induced pneumonitis mouse model. Furthermore, TO, but not CO, decreases MPO activity, as a parameter of inflammatory cell activation as well. In contrast, LO (with citronellal and geraniol as main components) aggravates airway responsiveness and MPO activity.

These findings are supported by earlier data showing the anti-inflammatory potential of TO with different compositions in other airway inflammation models. Thyme and ivy extracts significantly reversed leukocyte infiltration in a similar LPS-induced acute lung inflammation rat model [23]. In an ovalbumin-induced chronic asthma mouse model thymol exerted a significant anti-inflammatory effect by inhibiting inflammatory cell accumulation and reducing pro-inflammatory cytokine concentrations, as well as ameliorating airway hyperresponsiveness and histopathological alterations in the lungs [12]. In a carrageenan-induced pleurisy rat model TO (with carvacrol as main component) and carvacrol, but not thymol reduced leukocyte migration. However, both thymol and carvacrol had a potent anti-edema effect [9]. We also found that TO significantly reduced edema formation, but not inflammatory cell infiltration. This virtually contradictory result could be explained by the different composition of TO used in our study with thymol and *p*-cymene as main components and only 3.2% carvacrol content. Recent data demonstrated the anti-inflammatory effect of *p*-cymene as well, significantly alleviating LPS-induced acute lung inflammation, edema, TNF-*α*, IL-1*β* and IL-6 production [10,11].

There is abundant literature on the anti-inflammatory effect of cinnamaldehyde, the main component of CO, but these studies did not investigate functional parameters. It has been described that cinnamaldehyde inhibits the IL-1*β* and TNF-*α* production of macrophages stimulated by LPS in vitro [7], as well as in systemic inflammatory response syndrome in vivo [14]. The EO of Rimulus cinnamon or Guizhi (75.26% cinnamaldehyde) also decreased IL-1*β*, IL-18, IL-5, IF-*γ*, MCP-1, CSF-1 and MIP-1*β* in the serum, neutrophil cell count and nitric oxide production in the lung after intraperitoneal LPS administration [24].

LO with citral as main constituent has been described to suppress neutrophil adhesion and activation [25], edema formation and histopathological changes, as well as pro-inflammatory cytokine levels [13]. The anti-inflammatory property of citral is also supported by its ability to suppress LPS-induced cyclooxygenase-2 expression in the human macrophage-like U937 cell line [26]. In another in vitro study with macrophages LO pre-treatment in low doses significantly elevated IL-6 and IL-1*β* production; moreover, LO was only able to inhibit LPS effect in high doses. However, its main component, citral, effectively reduced cytokine levels both in pre-treatment and after LPS activation. These findings highlight the anti-inflammatory effect of the citral component of LO [8]. LO used in this study contained low levels of citral (neral + geranial), which can explain our results not supporting these earlier findings. However, much fewer data are available regarding the *Cymbopogon nardus* oil used in our study with citronellal and geraniol as main components. The EO of *Citrus maxima* (syn. *Citrus grandis*, contains citronellal and citronellol) and citronellal have been described to inhibit 5-lipoxygenase activity [27]. Geraniol, the other main component of LO used in our study, reduced MPO activity and pro-inflammatory cytokines production in vivo in an LPS-evoked acute lung injury model [15]. Moreover, geraniol dramatically reduced the expression of Toll-like receptor 4 and prevented nuclear factor-*κ*B activation [15]. Our contradictory findings might also be due to the different EO composition. Limonene, for instance, a minor component in *Cymbopogon nardus* was used in our study and has been described to induce bronchoconstriction in mice [28], which could explain the increased hyperresponsiveness elicited by our EO.

Therefore, when using the complex EOs instead of the individual components, the composition can significantly influence their biological effects and all components can interact with each other. For instance, 1,8-cineol, the minor TO and CO component, has some bronchodilator effects in guinea-pigs and rats [29,30], which can contribute to the improvement of respiratory parameters. The anti-inflammatory effect (steroid-sparing effect) and mucolytic potential of 1,8-cineol have also been proven in humans [31]. Myrtol, a standardized mixture of EOs containing cineole, limonene (minor components of CO and LO) and *α*-pinene (minor component of TO and CO) inhibited LPS-induced neutrophil accumulation, TNF-*α*, IL-6 and MPO activity increase in mice [32]. Furthermore, linalool, the minor component of TO and CO, attenuated histopathological changes and cytokine production in a similar model [21].

Airway inflammation is commonly accompanied by bacterial infections, therefore, the antibacterial and antifungal effects of EOs can also be beneficial in these conditions, particularly considering the spread of multiresistant bacteria and fungi [33,34,35,36,37]. We previously demonstrated the bactericidal effects of the vapor phase of TO, CO and LO, similarly to clove and eucalyptus EOs; as well as the bacteriostatic properties of Scots pine and peppermint EO against bacteria most common in respiratory infections including *Staphylococcus aureus*, methicillin-resistant *S. aureus* (MRSA) and *Pseudomonas aeruginosa* [38].

Despite the fact that our results provide useful information regarding the potential benefits and risks of EO inhalation in airway inflammation, a limitation of our experimental design is that we could not measure the exact EO concentration in the inhalation box but could only calculate its maximum value.

Considering the well-documented agonistic effects of thymol, cinnamaldehyde, carvacrol and linalool on members of the Transient Receptor Potential (TRP) ion channel family [39,40], the effects of these compounds might potentially be mediated by the Vanilloid 1 (TRPV1) or Ankyrin 1 (TRPA1) ion channels. TRPA1 and TRPV1 are expressed and often co-localized on the capsaicin-sensitive peptidergic afferents densely innervating the lung [41], as well as on pulmonary epithelial cells lymphocytes and bronchial smooth muscle cells [42,43]. TRPA1 and TRPV1 have been reported to be protective against the LPS-induced acute lung injury model [44,45,46]. The potential interactions of these essential oil components with the TRP ion channels in the LPS-induced acute lung injury model might open future drug developmental perspectives worth investigating.

## 4. Materials and Methods

### 4.1. EO Samples and the Gas Chromatographic Analysis of Their Composition

Cinnamon bark (*Cinnamomum zeylanicum* Nees. syn. *Cinnamomum verum* J.Presl), thyme (*Thymus vulgaris* L.), and lemongrass (*Cymbopogon nardus* (L.) Rendle) essential oils were bought from Aromax Ltd. (Budapest, Hungary). Gas chromatography–mass spectrometry (GC-MS, Agilent 6890 N/5973 N GC-MSD, Santa Clara, CA, USA) was used to analyze the chemical composition of the EO samples. The percentage composition of the EOs was evaluated with a flame ionization detector (FID). Compounds were identified based on retention times and spectral data of standard compounds, and the NIST 05 mass spectral library was also applied as previously described [38,47].

### 4.2. Animals

In the animal experiments, 10–18 week old female C57BL/6J mice [22] were used weighing 20.8 ± 0.26 g (Mean ± SEM) at the beginning of the experiment (Table S1). In order to avoid age-related differences, the age distribution of the mice was similar in each group. They were bred and kept in the Laboratory Animal House of the Department of Pharmacology and Pharmacotherapy at the University of Pécs. Optimal parameters were provided for all the animals (e.g., 325 × 170 × 140 mm cages, 12 h light/dark cycle, 24–25 °C, mouse chow, water).

During the experiments, the following regulations were taken into account: European legislation (Directive 2010/63/EU) and Hungarian Government regulation (40/2013., II. 14.) on the protection of animals used for scientific purposes, and the recommendations of the International Association for the Study of Pain. The study design was approved by the Ethics Committee on Animal Research of the University of Pécs (license No.: BA02/2000-26/2018, 21 June 2018).

### 4.3. Induction of Acute Airway Inflammation and Groups of Animals

Animals received *Escherichia coli* (serotype: 083) LPS intratracheal (i.t.) (100 µg LPS dissolved in 60 µL phosphate-buffered saline (PBS)) to induce acute airway inflammation. The endotoxin was isolated and purified in the Department of Microbiology, University of Pécs. During the administration of LPS, the animals were put under ketamine (100 mg/kg i.p.) and xylazine (5 mg/kg i.p.) anesthesia. Control mice received the same volume of sterile PBS [25]. Animals were treated with EO inhalation 1 h prior to and 4 and 23 h following LPS/PBS administration. Paraffin oil (PO) was used as negative control. Two oil drops (0.05 mL) were applied to a filter paper taped to the top of a box (33.5 × 19 × 12 cm) where mice were placed during the 30-min-long inhalation periods. The maximum concentration of the EOs was calculated as 6.55 µL/L. Mice were randomized into eight groups: (1) the control group treated with PBS i.t. and PO inhalation, (2) mice treated with LPS i.t. and PO inhalation, (3) PBS i.t. and TO inhalation, (4) LPS i.t. and TO inhalation, (5) PBS i.t. and CO inhalation, (6) LPS i.t. and CO inhalation, (7) PBS i.t. and LO inhalation, and (8) LPS i.t. and LO inhalation (*n* = 8–10/group).

### 4.4. Pulmonary Function Measurement

Respiratory functions were determined by unrestrained whole-body plethysmography (WBP) (PLY3211, Buxco Europe Ltd., Winchester, UK) in conscious and spontaneously breathing animals 24 h after PBS/LPS administration [48]. Baseline measurements were registered for 5 min with aerosolized saline to determine the respiratory parameters, such as breathing frequency, tidal volume, minute ventilation, time of inspiration and expiration, peak inspiratory and expiratory flow, and relaxation time after 2 min of acclimation period. Enhanced pause (Penh), a calculated parameter ((expiratory time/relaxation time)/(max. expiratory flow/max. inspiratory flow)) correlating with airway hyperresponsiveness [48] was measured for 15-15 min after bronchoconstriction evoked by increasing concentrations (11 and 22 mm) of aerosolized carbachol (carbamyl choline; Sigma, St. Louis, MO, USA; dissolved in saline, 50 μL per mouse). Airway function parameters were registered every 10 s and averaged by the BioSystem XA Software for Windows (Buxco Research Systems, Wilmington, NC, USA).

### 4.5. Histopathological Assessment and Semiquantitative Scoring

Mice were anaesthetized, and their lungs were harvested after airway function measurements. Left lungs were fixed in 4% formaldehyde, embedded in paraffin, sectioned with a microtome (5–7 μm), and stained with hematoxylin-eosin, as well as periodic acid–Schiff reaction for the visualization of the mucus-producing goblet cells. Inflammatory alterations were assessed by an expert pathologist in a blinded manner with semiquantitative histopathological scoring. Scoring parameters included the extent of perivascular edema (0–3), perivascular/peribronchial neutrophil accumulation (0–3), infiltration of activated macrophages/mononuclear cells in the alveolar spaces (0–2), and goblet cell hyperplasia of the bronchioles (0–2), as previously described [49,50]. A total inflammatory score was also assessed ranging between 0 and 10 by adding the subscores of the individual histopathological parameters.

### 4.6. Spectrophotometric Measurement of Myeloperoxidase (MPO) Activity

The activity of MPO enzyme correlates with the inflammatory infiltration of neutrophils and macrophages and this biochemical marker refers to the inflammation. Spectrophotometric measurement was made from the lung homogenates using H_2_O_2_-3,3′,5,5′-tetramethylbenzidine (TMB/H_2_O_2_), and the MPO activity was compared to a human standard MPO preparation as described earlier [48]. All reagents were obtained from Sigma-Aldrich Ltd. (St. Louis, MO, USA).

### 4.7. Statistical Analysis of Data

Respiratory parameters and MPO activity are expressed as mean ± SEM and analyzed with one-way ANOVA followed by Tukey post-test. Penh was analyzed with two-way ANOVA followed by Tukey post-test. Composite histopathological inflammatory score values were evaluated by Kruskal–Wallis analysis followed by Dunn’s post-test. In all cases, *p* < 0.05 was accepted as significant. Statistical analysis was performed in GraphPad Prism v6 software.

## 5. Conclusions

We conclude that the LPS-induced acute lung inflammation mouse model is an appropriate mechanism model to investigate the effect of EO inhalation. TO and CO can be considered as potential adjuvant treatment in airway inflammation, but the Ceylon type of LO use should be avoided.

## Figures and Tables

**Table 1:** The chemical composition of the investigated essential oils in percentage from 3 parallel measurements (main components in bold).

Compound	tR (min)	Thyme (%)	Cinnamon (%)	Lemongrass (%)
*α*-Pinene	5.8	0.9	0.9	−
Camphene	6.1	0.9	−	−
*β*−Myrcene	6.3	0.7	−	
*β*−Pinene	6.7	1.4	0.5	−
*α*−Terpinene	7.0	2.2	−	−
*o*-Cymene	7.0	0.3		
*p*-Cymene	7.4	**22.1**	1.2	−
Limonene	7.5	−	1.4	3.6
1,8-Cineole	7.9	9.8	2.1	−
*γ*-Terpinene	8.6	0.3	−	−
Linalool	10.2	5.1	3.9	−
Citronellal	10.4	−	−	**36.2**
Terpinen-4-ol	11.7	0.6	-	−
α-Terpineole	11.8	0.2	1.6	−
Citronellol	11.9	−	−	13.6
Camphore	12.1	0.5	−	−
Neral	12.2	−	−	1.0
Geraniol	12.4	−	−	**25.3**
Borneol	12.4	1.5	−	−
Geranial	12.8	−	−	2.2
Cinnamaldehyde	12.9	−	**74.0**	−
Anethol	13.1	−	4.5	−
Thymol	13.2	**46.3**	−	−
Carvacrol	13.3	3.2	−	−
Citronellyl acetate	14.1	−	−	2.3
Eugenol	14.2	−	2.7	−
Geranyl acetate	14.5	−	−	2.6
*β*-Elemene	14.7	0.4	−	2.9
*β*-Caryophyllene	15.4	2.5	1.7	−
*α*-Humulene	15.6	0.5	−	−
*β*-Cubebene	15.7	−	−	2.9
Cinnamyl acetate	15.8	−	5.3	−
α-Muurolene	16.6	−	−	1.6
*β*-Cadinene	16.9	−	−	3.2
Elemol	17.4	−	−	2.4
Caryophyllene oxide	18.0	0.5	−	−
Eudesmol	18.3	−	−	2.3
Total		99.5	99.8	99.8

## Supplementary Materials

The Supplementary Materials are available online. Figure S1: Percentage changes of respiratory parameters measured by unrestrained whole body plethysmography, Figure S2: Tidal volume and minute ventilation normalized to body weight, Table S1: Body weights of the mice.
