# Supplementary material for: Effects of Thymus vulgaris L., Cinnamomum verum J.Presl and Cymbopogon nardus (L.) Rendle Essential Oils in the Endotoxin-induced Acute Airway Inflammation Mouse Model
Source: Molecules. 2020 Aug 4;25(15):3553. doi: 10.3390/molecules25153553 (PMC7436258; doi:10.3390/molecules25153553)
Supplement: Supplementary file 1 [file molecules-25-03553-s001.pdf]

Supplementary Material

Effects of *Thymus vulgaris* L., *Cinnamomum verum* J.Presl and *Cymbopogon nardus* (L.) Rendle essential oils in the endotoxin-induced acute airway inflammation mouse model

Eszter Csikós[1,+], Kata Csekő[2,3+], Amir Reza Ashraf[1], Ágnes Kemény[2,3,4], László Kereskai[5], Béla Kocsis[6], Andrea Böszörményi[7], Zsuzsanna Helyes[2,3,8], Györgyi Horváth[1,*]

[1] Department of Pharmacognosy, Faculty of Pharmacy, University of Pécs, H-7624, Pécs, Hungary
[2] Department of Pharmacology and Pharmacotherapy, Medical School; University of Pécs, H-7624, Pécs, Hungary
[3] Szentágothai Research Centre, University of Pécs, H-7624, Pécs, Hungary
[4] Department of Medical Biology and Central Electron Microscope Laboratory, Medical School, University of Pécs, H-7624, Pécs, Hungary
[5] Department of Pathology, Medical School, University of Pécs, H-7624, Pécs, Hungary
[6] Department of Medical Microbiology and Immunology, Medical School, University of Pécs, H-7624, Pécs, Hungary
[7] Department of Pharmacognosy, Faculty of Pharmacy, Semmelweis University, H-1085, Budapest, Hungary
[8] PharmInVivo Ltd, H-7629, Pécs, Hungary
* Correspondence: horvath.gyorgyi@gytk.pte.hu; Tel.: +36-72-503650-28823
[+] The authors contributed equally to the present work.

Figure S1. Percentage changes of respiratory parameters measured by unrestrained whole-body plethysmography

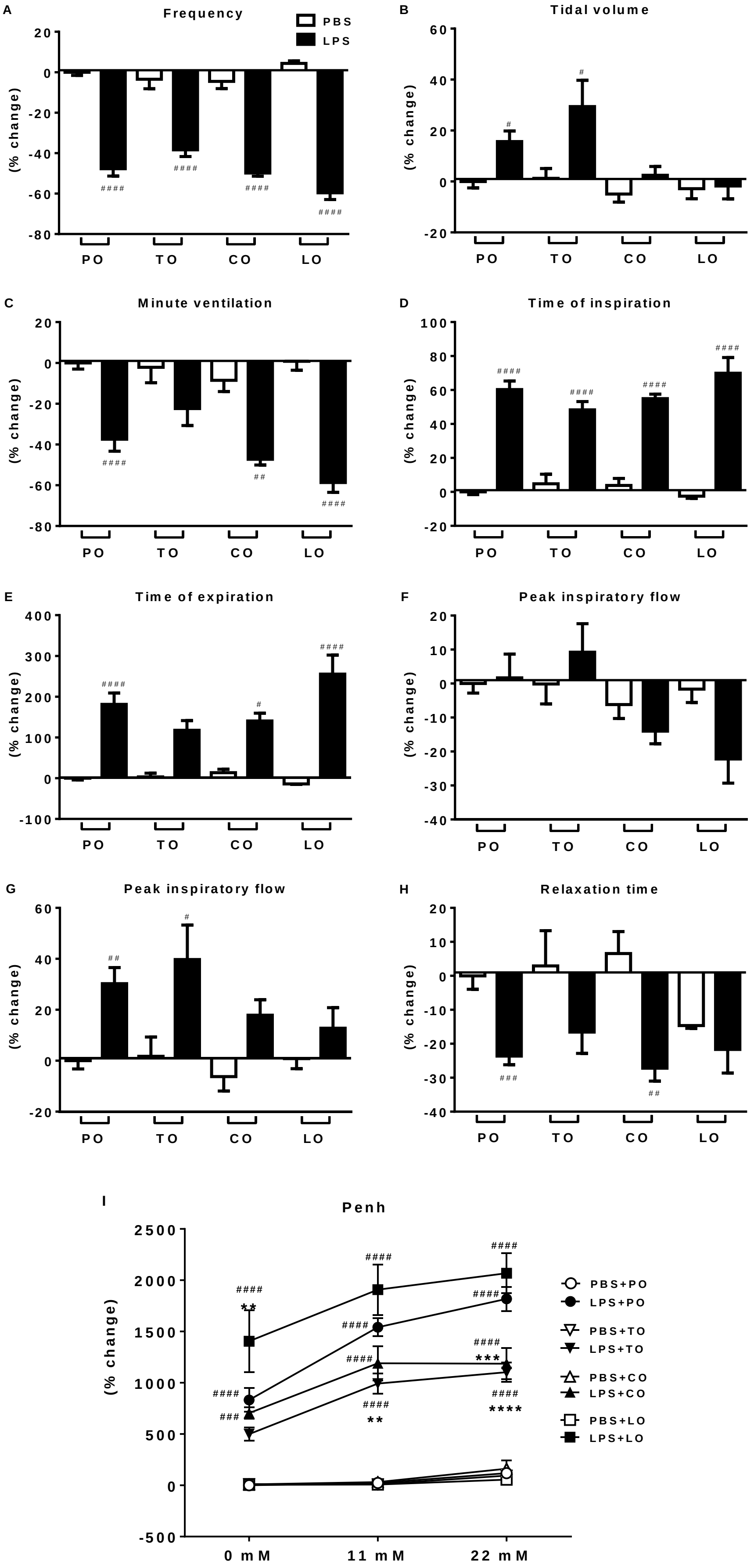

Figure S1. Effects of thyme (TO), cinnamon (CO) and lemongrass essential oil (LO) on respiratory parameters after LPS/PBS treatment compared to paraffin oil (PO) and PBS treatment as control. (n = 8-10 / group, #$p < 0.05$, ##$p < 0.005$, ###$p < 0.0005$, ####$p < 0.0001$ vs. respective PBS-treated group, *$p < 0.05$, **$p < 0.005$, ***$p < 0.0005$, ****$p < 0.0001$ vs. LPS-PO-treated mice)

Figure S2. Tidal volume and Minute ventilation normalized to body weight

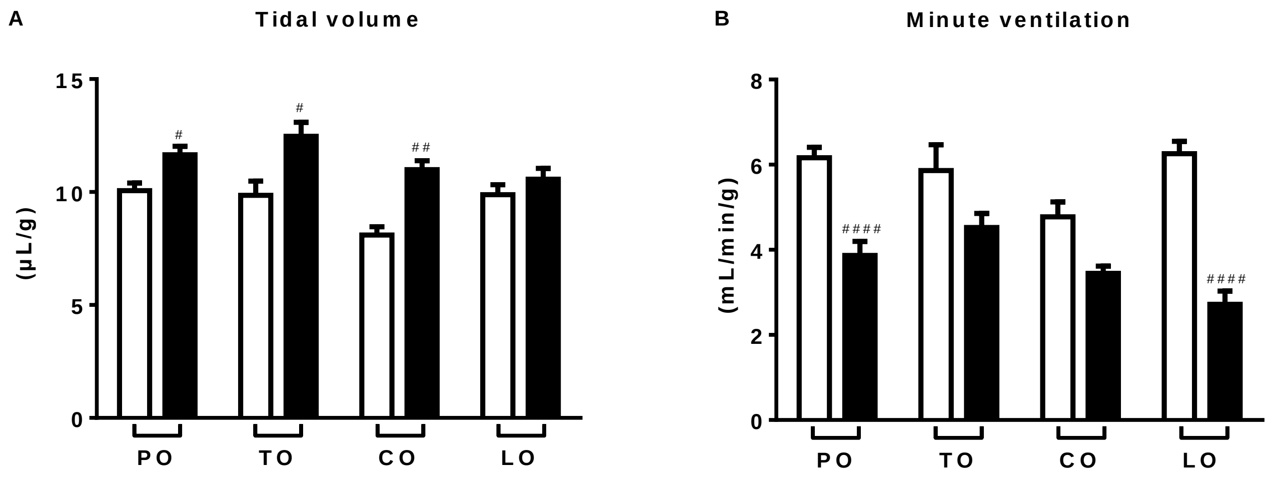

Figure S2. Effects of thyme (TO), cinnamon (CO) and lemongrass essential oil (LO) on (**A**) tidal volume and (**B**) minute ventilation compared to paraffin oil (PO) after LPS/PBS treatment. (n = 8-10 / group, #p < 0.05, ##p < 0.005, ###p < 0.0005, ####p < 0.0001 vs. respective PBS-treated group)

Table S1. Body weights of the mice

Table S1. Body weights of the mice.

**Group**	**0h Mean±SEM (g)**	**24h Mean±SEM (g)**
PBS+PO	19.5±0.38	19.4±0.41
LPS+PO	21.7±0.48	19.4±0.39
PBS+TO	19.2±0.75	19.2±0.70
LPS+TO	20.5±0.22	18.0±0.26
PBS+CO	20.1±0.70	20.1±0.70
LPS+CO	22.6±1.56	20.1±1.32
PBS+LO	22.7±0.61	22.9±0.55
LPS+LO	20.5±0.42	18.1±0.40
